# Management of Spontaneous Chylothorax in a Neonate: A Medical Challenge

**DOI:** 10.7759/cureus.12942

**Published:** 2021-01-27

**Authors:** Irfan Ullah, Shahzad Rauf, Jahanzeb Malik, Kiran Shafiq Khan, Abdul Wali Khan

**Affiliations:** 1 Internal Medicine, Kabir Medical College, Peshawar, PAK; 2 Pediatrics, Khyber Medical University, Peshawar, PAK; 3 Cardiology, Rawalpindi Institute of Cardiology, Rawalpindi, PAK; 4 Internal Medicine, Dow University of Health Sciences, Karachi, PAK; 5 Internal Medicine, College of Physician and Surgeons Pakistan, Peshawar, PAK; 6 Internal Medicine, Hayatabad Medical Complex Peshawar, Peshawar, PAK

**Keywords:** chylothorax, neonate

## Abstract

Chylothorax or collection of lymphatic fluid in the pleural space is an exceedingly rare cause of pleural effusion and respiratory distress in neonates. In adults, prompt diagnosis and effective treatment regimen can aid in the resolution of chylothorax; however, in neonates treating the condition can be an onerous challenge for the pediatricians due to the rarity and perplexing clinical presentation. We elucidate a case of spontaneous chylothorax in a 15-day-old neonate who presented to us with respiratory distress, chest indrawing, and cyanosis for nine days. The prenatal and birth history of the patient were insignificant. The detailed laboratory and radiological assessment of the patient divulged a diagnosis of spontaneous chylothorax for which the patient was intubated. Unfortunately, the patient deteriorated and eventually expired on the 23rd postnatal day due to the complications of respiratory acidosis and hypoxemia.

## Introduction

Chylothorax is defined as the collection of lymphatic fluid in the pleural space. It commonly causes large pleural effusions in adults, but it rarely causes respiratory distress in newborns [1,2]. It can be unilateral or bilateral in origin secondary due to trauma [3]. The incidence of chylothorax in neonates is one in 20,000 live births with a mortality rate of 64% [4]. This trauma can be a result of thoracic or cardiovascular surgery, and in some patients, it is associated with lymphangiomatosis. The right lung is more frequently involved and clinically, the patient presents with respiratory distress and diminished breath sounds on the affected side [4]. The prognosis is usually favorable; however, resolution can be protracted in severe cases with treatment depending upon the size and amount of the collected chyle [2,4]. We present a case of spontaneous chylothorax in a newborn infant. The diagnostic evaluation and appropriate management proved a medical challenge.

## Case presentation

A 15-day-old female neonate presented to us with breathing difficulty and cyanosis for nine days after a referral from the district hospital. On examination, respiratory rate was 74 breaths/min, marked chest in-drawing, right-sided thoracic bulge with decreased air entry, and stony dull percussion. According to her mother, she was her second child, born in the hospital through normal vaginal delivery. She gave the history of oligohydramnios during the end of the second trimester, diagnosed on routine ultrasound. Otherwise, the delivery was uneventful except delayed cry, appearance, pulse, grimace, activity, and respiration (APGAR) score 6/10 at birth, and cord around the neck. The infant developed breathing difficulty first on the sixth and 12th day of her life after which she was referred and admitted to our tertiary hospital where oxygen saturation was maintained and she was started on intravenous fluids.

Owing to the deteriorating condition of the patient, a laboratory workup for sepsis was done and intravenous antibiotics including Ampicillin (200 mg/kg/day in two divided dose), Amikacin (15 mg/kg/day in two divided dose), Ceftazidime (150 mg/kg/day in two divided dose), and Vancomycin (45 mg/kg/day in three divided dose) were administered. The results of the complete blood picture on the first, second, and third day of admission are elucidated in Table 1 and other baseline lab parameters are shown in Table 2.

**Table 1:** A complete blood count of the patient on the first, second, and third day of admission HCT, hematocrit; MCV, mean corpuscular volume; MCH, mean corpuscular hemoglobin; MCHC, mean corpuscular hemoglobin concentration; MPV, mean platelet volume.

Variables	Normal	First day	Second day	Third day
White Blood Cell	4-11 × 10^3^/µl	22.8	21.2	10.2
Red Blood Cell	4-6 × 10^6^/µl	5.79	5.26	6.9
Hemoglobin	11.5-17.5 g/dl	18.9	16.7	21.5
HCT	36%-54%	54.7	45	66.1
MCV	76-96 fl	94.4	85.5	95.7
MCH	27-33 pg	32.7	31.7	31.2
MCHC	33-35 g/dl	34.7	37.1	32.6
Platelets	150-450 × 10^3^/µl	469	342	147
MPV	7.2-11 fl	9.6	7.5	10.9
% Neutrophil	40%-75%	72.4	77.8	80.5
% Lymphocyte	20%-45%	20	17.1	13

**Table 2:** Baseline lab parameters LDL, low-density lipoproteins; HDL, high-density lipoproteins; SGPT, serum glutamic pyruvic transaminase

Variables	Normal	Patient Result
Triglycerides	< 200 mg/dl	96
Cholesterol	< 200 mg/dl	123
LDL Cholesterol	< 150 mg/dl	74
HDL Cholesterol	35-65 mg/dl	27
Creatinine	0.3-0.7 mg/dl	0.12
S. Bilirubin	0.2-1.0 mg/dl	0.48
SGPT	< 31 U/L	19
Alkaline Phosphatase	< 727 U/L	176
Blood Urea	05-45 mg/dl	16
Serum Calcium	7.6-10.4mg/dl	7.9
Sodium	136-149 Meq/L	133
Potassium	3.8-5.2 Meq/L	4.0
Chloride	96-110 Meq/L	1.3

**Figure 1:**
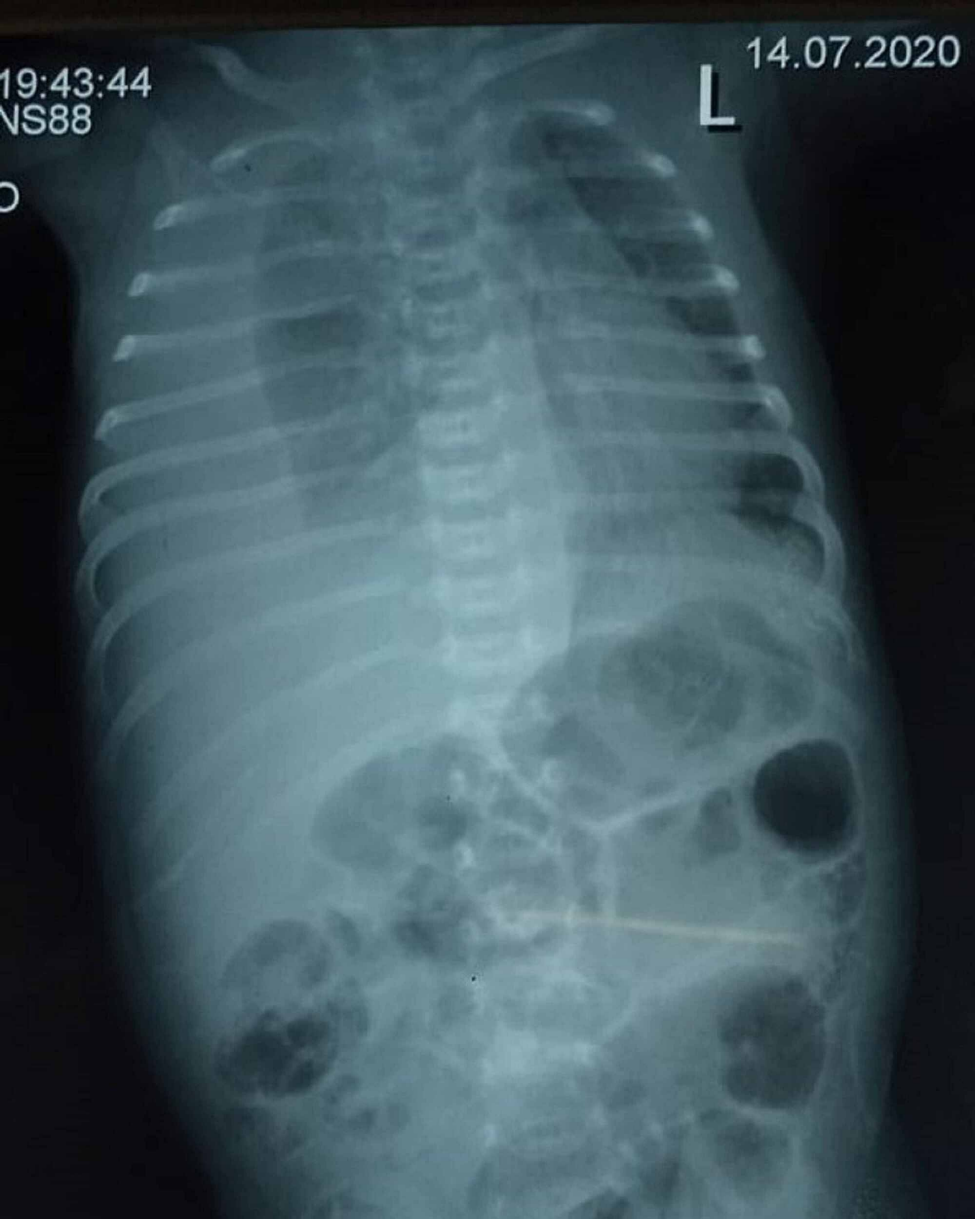
Chest X-ray before pleural tap showing massive right-sided pleural effusion

**Figure 2:**
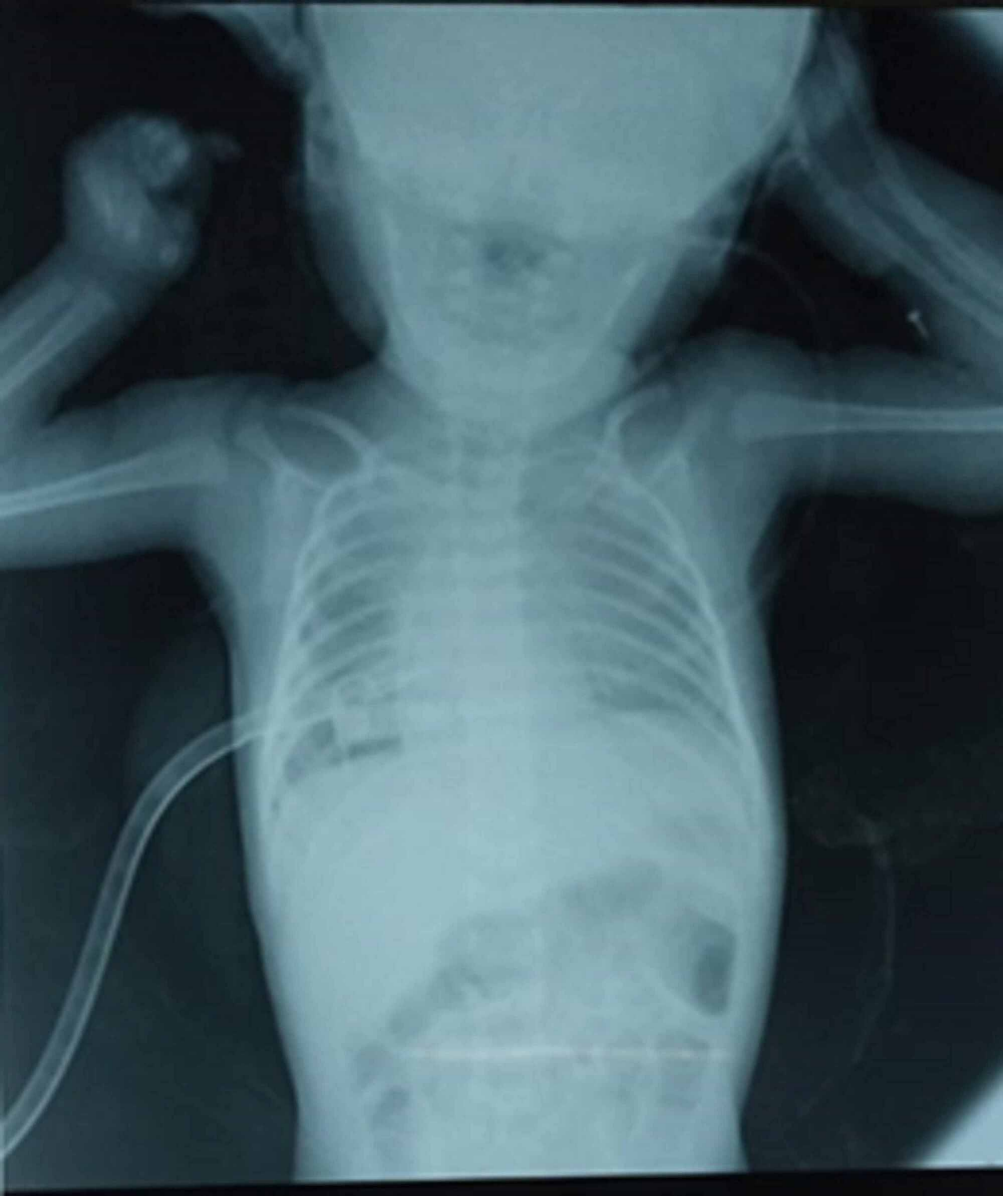
Chest X-ray after pleural tap via chest tube

Chest X-ray revealed right hemithorax with a mediastinal shift to the left side. No cystic lesion was noted on X-ray; therefore, it excluded the Bochdalk Hernia (on the left side) and Morgani Hernia (on the right side). Figures 1, 2 show a chest X-ray before and after pleural tap, respectively.

**Figure 3:**
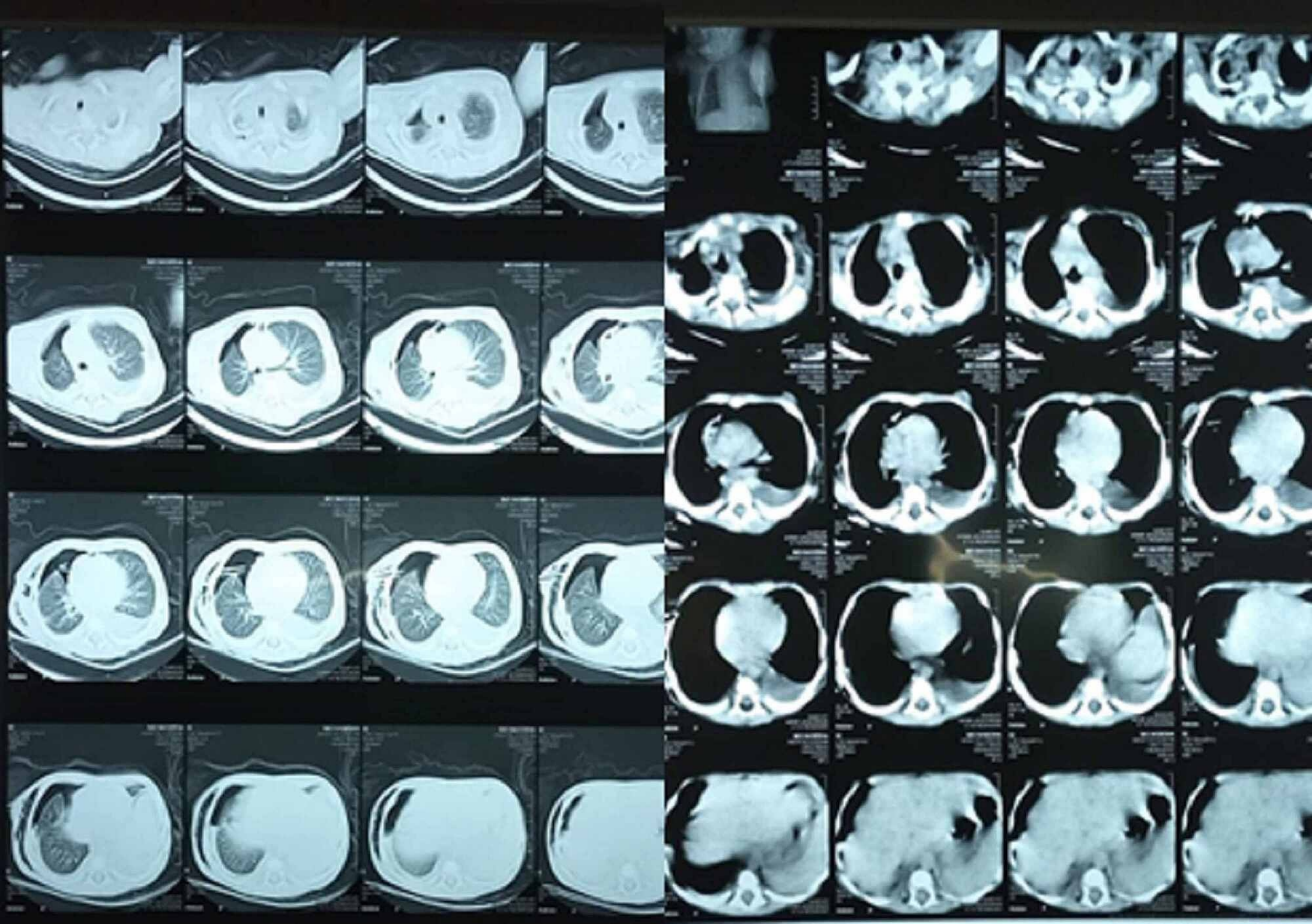
Computed tomography chest showing bilateral pleural effusion, subcutaneous emphysema, and pneumothorax.

The pleural fluid analysis showed protein 2.6 g/dl, total cell count 680/mm, red blood cell 10-15/HPF, neutrophil 40%, lymphocyte 60%, no gram staining, and acid-fast bacilli (AFB) was noted. The second pleural tab revealed greenish fluid suggesting empyema. Chest intubation drained around 250 ml fluid. Computed tomography (CT) showed bilateral pleural effusion, pneumothorax, and subcutaneous emphysema on the right side while moderate pleural effusion was noted on the left side (Figure 3).

Based on the above investigations, a diagnosis of neonatal spontaneous chylothorax was made. Arterial blood gas (ABG) showed respiratory acidosis and hypoxemia. The infant was intubated, and even after mechanical ventilation, the respiratory rate got worsened. The condition of the neonate continued to deteriorate and she expired on the 23rd postnatal day.

## Discussion

The literature review showed 36 cases of pleural effusion in neonates of non-traumatic origin [5]. Out of all, 18 cases had chylous fluid, while in seven cases clear and straw-colored was observed. In three cases, the fluid was initially clear, but re-accumulated and later taps drew milky fluid [2,5]. Chylothorax is a life-threatening condition that may result in metabolic, nutritional, and immunological complications [6]. It is one of the common causes of pleural effusion that may lead to respiratory distress in neonates [1,6]. The proportion of fats, immune cells, and proteins within chyle fluid are similar to serum. The survival rate of neonates with chylothorax varies according to the amount of fluid and is highly dependent on other co-morbidities [4]. Management approach varies among different neonatal setups and treatment options range from diet modification to medication or surgical intervention [6]. It has been proposed that the development of the lymphatic system begins early during the fifth week of fetal life [2]. The thoracic duct is a major lymphatic channel to cross the posterior mediastinum at the level of T5. Abnormalities or injury to the thoracic duct below the level of T-5 result in right-sided chylothorax, or if it is above T-5, it may lead to a left-sided effusion, which is very rare [7]. It is also assumed that persistent pleural effusion is because of tear/injury to the thoracic duct during the birth process [3].

The diagnosis was made on the character of aspirated fluid. Unlike our laboratory investigation results, triglycerides and total lipid levels were higher than those measured in plasma, whereas the cholesterol levels were lower than normal. A sizeable amount of protein was also present and numerous cells, predominantly lymphocytic cells, were found. Likewise, hypoproteinemia, hyponatremia, and metabolic acidosis need to be replaced [2]. It has been observed that feeding with formula milk containing long-chain fatty acids increases intestinal lymph flow and leads to persistent pleural effusions because fatty meal increases the basal flow of lymph in the thoracic duct by 10-folds [7]. Protein and carbohydrate cause smaller increases in lymph flow [2]. Medium and short-chain fatty acid bypass the lymph flow and directly absorb into the portal vein system [7]. In previously reported cases, death has been reported due to starvation; therefore, the mainstay for the treatment of severe cases should be intravenous nutrients in the present times that replace the deficient nutrients [8]. Parenteral feeding via a peripheral vein is recommended in infants with chylothorax to prevent superior vena cava thrombosis [7,8]. Surgery should be reserved as a last alternative until the trial of parenteral feeding fails to yield favorable outcomes [2].

In our patient, there was no clear cut indication of trauma; however, the clinical course proposed the probability of an injury to the thoracic duct during labor or at the time of delivery. A lack of cyanosis and respiratory distress immediately after birth seemed to rule out the congenital defect of the duct. Respiratory distress and cyanosis were noted on the sixth day of the postnatal period, although chest ultrasound demonstrated a large amount of fluid in pleural space. Initially, on the first chest X-ray, sepsis with right-sided spontaneous pleural effusion was suspected. However, this infant was noted to have a second episode of marked respiratory distress on the 12th day after birth. After evaluation of milky pleural fluid and an early examination of the cellular components of the pleural fluid, including a lymphocyte count, can aid in the diagnosis. Unlike other cases reported in the literature [2], our patient was in critical condition and developed severe respiratory distress, and expired on the 23rd postnatal day. Since intrauterine distress is relatively common and chylothorax in a newborn infant is rare, other unknown factors must be involved in the etiology and should be deliberate which remains obscure.

## Conclusions

The late presentation of spontaneous chylothorax in a neonate is a rare entity and its medical management is an exacting challenge. There is an unmet need for curation of guidelines that consider a multidisciplinary approach for appropriate management of the condition. Further studies are needed to ascertain the etiology of neonatal chylothorax.
